# Medication error and associated factors among adults admitted to emergency ward at the university of Gondar comprehensive specialized hospital, North-West Ethiopia: a cross-sectional study, 2022

**DOI:** 10.1186/s40545-023-00616-2

**Published:** 2023-11-17

**Authors:** Saron Naji Gebremariam, Fasiel Dula Sema, Abdisa Gemedi Jara, Banchamlak Teferi Mekonen, Gizework Alemnew Mekonnen

**Affiliations:** Department of Clinical Pharmacy, School of Pharmacy, College of Medicine and Health Sciences, University of Gondar, Gondar, Ethiopia

**Keywords:** Medication error, Patient safety, Adverse drug event

## Abstract

**Background:**

Medication errors are the most common cause of preventable adverse drug events at the emergency ward.

**Objectives:**

This study assessed medication errors and associated factors among adult patients admitted to the emergency ward at the University of Gondar Comprehensive Specialized Hospital, North-West Ethiopia.

**Methods:**

A cross-sectional study was conducted from June 1, 2022, to August 30, 2022. Data were entered into EpiData Manager 4.6.0.0 for clearing and exported to SPSS version 24 for analysis. Descriptive statistics such as frequencies, medians with an interquartile range and inferential statistics like binary logistic regression were used for data analysis. The level of significance was declared at a *p* value less than 0.05 with a 95% confidence interval.

**Results:**

From 422 study participants, medication errors were found in three-fourths (74.4%) of study participants. The most frequent type of medication error was omitted dose (26.27%). From a total of 491 medication errors, 97.75% were not prevented before reaching patients. More than one-third (38.9%) of medication errors had potentially moderate harmful outcomes. More than half (55.15%) of possible causes of medication errors committed by staff are due to behavioral factors. Physicians accepted 99.16% and nurses accepted 98.71% of clinical pharmacist intervention. Hospital stay ≥ 6 days (AOR: 3.00 95% CI 1.65–5.45, *p* < 0.001), polypharmacy (AOR: 5.47, 95% CI 2.77–10.81 *p* < 0.001), and Charlson comorbidity index ≥ 3 (AOR: 1.94, 95% CI (1.02–3.68), *p* < 0.04) significantly associated with medication error.

**Conclusions:**

About three-fourths of adult patients admitted to the emergency ward experienced medication errors. A considerable amount of medication errors were potentially moderately harmful. Most medication errors were due to behavioral factors. Most clinical pharmacists’ interventions were accepted by physicians and nurses. Patients who stayed longer at the emergency ward, had a Charlson comorbidity index value of ≥ 3, and were on polypharmacy were at high risk of medication error. The hospital should strive to reduce medication errors at the emergency ward.

## Background

Patient safety in the healthcare has gained significant attention worldwide [1]. Currently, patients are continuously harmed while receiving health care services in both high- and low-income countries [2]. Patient safety is concerned with enhancing safe practice and minimizing adverse medical outcomes in any health care facility [3]. Health care delivery systems tend to be complex [4]. Health practitioners who operate in such systems are more likely to make medication errors [5]. Around the world, as many as four in ten patients using healthcare are harmed. The majority of them are medication related which could be preventable [6].

"A medication error is any preventable event that may cause or lead to inappropriate medication use or patient harm while the medication is under the control of the health care professional, patient, or consumer"[7].

The global patient safety challenge was launched by WHO in 2017. It was aimed at reducing medication errors and subsequent harm in healthcare settings by 50% [8]. Studies done in Africa revealed that around half of adverse drug events (ADEs) were due to medication errors (MEs) [9]. In Ethiopia, different healthcare settings have reported medication errors becoming more common [10, 11].

Despite the improvement of the health care system and practices, medication errors are still a problem that needs a global solution. WHO reports that 3.4–97% of adult patients have at least one medication error at admission [12]. In low-income countries, such as Africa, MEs are reported in 75–97% of patients [9].

Medication errors could cause tragic outcomes for patients, health care professionals, and health institutions. MEs must be addressed boldly and provided with a solution. Any failure in medication use processes could have devastating consequences for patients [13]. In clinical practice, making an error, especially with medications, is the most stressful professional experience. It is not possible to resolve such mistakes by apologizing or calming the loss since the damage may compromise or take a person’s life [14]. Health care professionals who commit MEs could feel guilty, shame, and self-doubt which affects them mentally and socially [15]. Healthcare institutions could face huge legal fees and settlement costs for mistakes. Such kinds of events could affect the hospital’s name and re-accreditation [16].

In addition to patient harm, MEs have a profound economic burden. Studies on the economic impact of MEs reveal that the mean cost (pre-error) per study ranges from €2.58 to €111,727.08 [17]. In the United Kingdom, the burden of MEs, including primary care costs for medication-related admissions and secondary care costs for prolonged hospitalization resulting from preventable ADE, is £ 98.5 million per year [18]. Despite interventions to reduce ME incidence, no difference in the number of patients hospitalized or dying from ME has been observed [11].

The present study was conducted on patients admitted to the adult emergency ward. The emergency ward is a place where the majority of medication errors are assumed to have occurred [19]. Even if there is one study about MEs on the emergency ward in Ethiopia, it is specifically limited to assessing the prevalence and distribution of prescription errors only. The present study, however, examined variables not well addressed in the previous study. These variables included the possible causes of ME, patient outcomes, types of medication-related interventions given, and acceptance rates for the interventions. Moreover, since the previous study was single-centered, extrapolating the findings to this study setting is difficult due to the possible heterogeneity of the study population and the study setting. Therefore, this study was aimed at assessing medication errors and associated factors among adult patients admitted to the emergency ward at the University of Gondar Comprehensive Specialized Hospital, North-West Ethiopia. The study may help the hospital and similar settings to develop strategies targeted at reducing risks and improving patient safety. Using this research, we can develop future interventions in our settings to prevent MEs.

## Methods

### Study design, area, and period

A cross-sectional study was conducted from June 1, 2022 to August 30, 2022G.C in the emergency ward at the UoGCSH, located in Gondar town, northwest Ethiopia. The calculated flying distance from Addis Ababa, the capital city of Ethiopia, to Gondar is equal to *262 miles,* which is equal to *421 km,* and the driving distance between Addis Ababa and Gondar is *727.22 km* [20]. The University of Gondar Comprehensive Specialized Hospital is a tertiary care facility with different wards, including emergency, ambulatory, pediatrics, oncology, gynecology, and surgery wards. According to the UoGCSH’s 2021/2022 annual report, around 280,000 patients visit the hospital and 12,000 are admitted to the emergency ward.

### Population, inclusion, and exclusion criteria

Patients aged ≥ 18 years admitted to the emergency ward at UoGCSH were the source population. However, those adult patients admitted to the emergency ward at UoGCSH during the study were the study population. Patients who had at least one medication order and stayed for at least 24 h on the emergency ward were included. Patients were excluded if they were too ill to answer interview questions and/or did not have a caregiver.

### Sample size determination and sampling procedure

The sample size was determined using a single population proportion formula with the assumption of a 95% confidence level, 5% margin of error, and 50% proportion. The prevalence of ME at the emergency ward was not known in Ethiopia. During the study, 50% population proportion was used:

$$n={z}^{2 }p(1-p)/{w}^{2}$$

*n* = (1.96)^2^(0.5 × 0.5)/ (0.05)^2^ = 384.2

By adding a 10% contingency (384.2*10% = 38) to the calculated sample size, 422 patients were estimated for the study. Where *n* = sample size, *p* = sample proportion/population proportion, z = confidence level/Z-score and ${w}^{2}$= margin of error.

A systematic random sampling technique was used to collect data from patients who fulfilled the inclusion criteria. It was estimated that 3000 patients would be admitted to the ward during the 3-month data collection period. The formula “*k* = *N*/*n*” was used to obtain the “*k*” value. As a result, *k* was set to "7," and the first patient admitted to the ward was chosen by lottery after rolling a piece of paper one through seven and randomly selecting one of the rolled papers. After the first patient was selected, every seventh patient admitted to the ward fulfilling the inclusion criteria was taken as a sample.

### Variables of the study

The dependent variable was medication error. The independent variables include gender, patients with uncorrected/uncorrectable visual impairment, patients with uncorrected/uncorrectable renal impairment, patients diagnosed with pneumonia, presence of comorbidity, Charlson comorbidity index, duration of patient stay in the emergency ward after admission, and number of medications the patient is using after admission.

### Operational definitions

Comorbidities: according to the center for disease control and prevention (CDC), comorbidities are defined as when a person has more than one disease or condition at the same time [21].

Patient harm: is any unintended and unnecessary harm resulting from, or contributed to by, health care [22].

Medication errors: are defined as “any preventable event that may cause or lead to inappropriate medication use or patient harm while the medication is in the control of the healthcare professional, patient, or consumer.”[23].

Near miss or “close call”: a prevented medicine-related patient safety incident that could have led to patient harm.

Adverse drug event (ADE): is an event that occurs when a medicine is administered to a person in order to improve their health but instead causes harm or exposes the person to potential harm. The occurrence of an ADE does not necessarily indicate an error or poor quality of care.

Preventable adverse drug events: are adverse drug events that result from a medication error that reaches the patient and causes any degree of harm.

Potential adverse drug events: are adverse drug events resulting from medication errors that do not cause any harm, either because they are intercepted before reaching the patient or because of luck [24].

The NCC MERP Index: the severity of MEs and ADEs can be assessed using the detailed scale published by the National Coordinating Council for Medication Error Reporting and Prevention (NCC MERP), which is categorized from A to I. Categories A through D of the NCC MERP Index are relevant to MEs; and Categories E through I of the NCC MERP Index are relevant to ADEs:

- Category A: Circumstances or events that have the capacity to cause error.
- Category B: An error occurred but the error did not reach the patient.
- Category C: An error occurred that reached the patient but did not harm the patient.
- Category D: An error occurred that reached the patient and required monitoring or intervention to confirm that it resulted in no harm to the patient and/or required intervention to preclude harm.
- Category E: An error occurred that resulted in the need for treatment or intervention and caused temporary patient harm.
- Category F: An error occurred that resulted in initial or prolonged hospitalization and caused temporary harm [25].

Prescription error: “a failure in the prescription writing process that results in wrong instructions about one or more of the normal features of a prescription. The ‘normal features’ include the identity of the recipient, the identity of the drug, the formulation, dose, route, timing, frequency, and duration of administration”[26].

Medication transcription error: “any discrepancy between the physician’s medication order and the medication order transcribed onto any document related to the patient concerned, such as the medical record, medication chart, medication request sheet, discharge medication chart, and/or any other similar document” [27]. In this study, when medication is written on the order sheet of the medical chart but not written on prescription paper for the patient or caregiver to buy or bring the medication, it is considered a transcription error.

Administration error: a failure in one of the nine “rights” of medication administration (right patient, medication, time, dose, route, documentation, action, form, and response) [28].

Monitoring error: failure to review a prescribed regimen for appropriateness and detection of problems or failure to use appropriate clinical or laboratory data for adequate assessment of the patient’s response to prescribed therapy [29].

“Omission of transcription”: when a medication written on an order sheet is not transcribed to a prescription paper (to allow the patient to bring and take the medication).

Dose omitted: a prescribed medication or dose that is already in the hands of the patient, caregiver, or nurse but is not given**.**

Actual severe harm: permanent harm experienced by the patient due to medication error.

Actual moderate harm: reversible harm experienced by the patient due to medication errors that require active treatment.

Actual mild harm: reversible harm experienced by the patient due to medication errors that require monitoring.

Potentially fatal harm: no harm has occurred, but the medication error could have an adverse outcome that could be fatal.

Potentially severe harm: no harm has occurred, but the medication error could cause permanent harm.

Potentially moderate harm: no harm has occurred, but active treatment is required to prevent harm that could be caused by a medication error.

Potentially mild harm: no harm has occurred, but monitoring is required to prevent harm that will be caused by a medication error.

“Start a drug”: is an intervention given by clinical pharmacists when a medication at hand is not started to be administered (by a nurse or by the patient himself/herself) or when a drug is written on the order sheet of the medical chart but not transcribed to prescription paper for the patient to bring and take the medication.

“Continue a drug”: is an intervention given by clinical pharmacists when the next dose to be administered is interrupted (missed) after administration has been started at a certain time.

Fully accepted: when a person accepts an intervention given by a clinical pharmacist without any doubt and acts immediately.

Partially accepted: when a person accepts an intervention given by a clinical pharmacist with some level of doubt and agrees to act based on that later point in time and/or partially act upon [30].

Not accepted: when a person does not accept an intervention provided by a clinical pharmacist.

### Data collection instrument, procedure, and quality control

To collect the data, three clinical pharmacists were trained on the data collection tool. At the emergency ward, each patient was followed from admission until discharge and assessed on their medication use process. Medication errors were identified in accordance with the “Standard Treatment Guidelines for General Hospitals,” third edition, published in 2014, Ethiopia, and a pharmacotherapy book were also employed [31, 32]. In addition, different variables regarding medication errors were identified using publications by WHO, “Reporting and Learning Systems of Medication Errors: The Role of Pharmacovigilance Centers” and “Medication Errors: Technical Series on Safer Primary Care [33, 34].

Part of the questionnaire that needed the response of patients or caregivers (the socio-demographic data) was translated into the local language (Amharic), and then they were interviewed. The “Amharic” version of the questions was back translated into the English version to confirm translation consistency. Clinical information was gathered from patient medical records (procedure notes, physician orders, prescription papers, medication administration records, physician progress notes, pertinent laboratory reports, and nursing progress notes). Data were also collected through direct observation of patients and health care professionals during the medication use process. The Charlson comorbidity index and estimated 10-year survival were assessed [35]. The eGFR of patients was calculated using the Cockcroft–Gault Equations [36]. For patients with renal impairment, “Drug Prescribing in Renal Failure” was used as a guide to determine the appropriateness of the drug dose prescribed [37].

Questioners used in the Swiss Sentinel Surveillance Network study were used to gather socio-demographic data, clinical characteristics of patients and the presence of ME in patients [38]. A checklist prepared for the California Health Care Foundation for tracking MEs in hospitals was used to collect medication order information and the categorization or staging of medication errors [39], and a model form for reporting medication errors, designed by WHO in the “Reporting and Learning System for Medication Errors: The Role of Pharmacovigilance Centers” guideline, was used to obtain types of ME, patient outcomes, and possible causes of ME [33]. The severity of ME was reported according to the detailed scale published by the National Coordinating Council for Medication Error Reduction and Prevention (NCC MERP) [25]. Data collectors intervened when medication errors occurred during data collection. Interventions given by clinical pharmacists were obtained from a study done in Italy [30]. In addition, the tool employed to measure the acceptance rate of the interventions were obtained from a study done in Switzerland [40].

After the questionnaire was pre-tested on 5% (21 individuals) of the study population, some rearrangements were made to the instrument to make it more favorable for data collection. The consistency of the responses was evaluated after data was obtained from the pretested questionnaire. Three clinical pharmacists were assigned to collect data. They were trained for 3 days on the study objectives and how to use the tool or questionnaire properly. Data collectors were supervised while collecting data. The data was checked for its completeness and consistency on a daily basis.

### Analysis of data

After data were entered, cleared, and checked with EpiData Manager 4.6.0.0, it was exported to Statistical Package for Social Sciences (SPSS) version 24 for analysis. The normality of the different variables included in the analysis was checked using histograms and the Shapiro–Wilk test. Descriptive statistics were used to characterize dependent and independent variables. The frequency and percentage of socio-demographic characteristics, clinical characteristics, medication-related characteristics, different variables that measure medication errors, the outcome of MEs, possible causes of MEs, medication-related interventions, and the rate of acceptance of the given interventions were performed. Categorical variables were described as frequency and percentages, and continuous variables as the median and interquartile range (IQR). Tables and figures were used to summarize and describe the results.

The “Enter method” is used for entering variables into the binary logistic regression **model.** All variables having a *p* value < 0.2 in bivariable binary logistic regression analysis were entered into multivariable binary logistic regression to test the strength of association between dependent and independent variables. Model fitness was checked using the Hosmer–Lemeshow goodness-of-fit test. The assumption of independence (adequacy of cells) was checked by chi-square statistics and the value obtained was (6.625). Only variables not violating the assumption were analyzed later by multivariable binary logistic regression. Prior to analysis, variables were tested for multicollinearity by the variance inflation factor (VIF). The included variables had VIF values in the range of 1–1.8 [41]. The presence of outliers was also checked using the inter-quartile range method (*Q*1–1.5*IQR and *Q*3 + 1.5*IQR) [42]. In the present study, no outliers were identified. The strength of the association was measured using an odds ratio (OR). A *p* value of < 0.05 was considered statistically significant with a 95% level of confidence.

## Result

### Patient demographic data

The median age of participants was 40 years (IQR: 28–55). More than half (54.5%) of the participants were female. Nearly three-fourths (72.5%) of patients were rural residents. Over half (52.6%) of patients are married. Only few a (3.1%) of patients have smoking habits. Less than half (45.3%) of patients use alcoholic drinks (Table 1).

**Table 1:** Socio-demographic characteristics of patients admitted to the emergency ward at UoGCSH

Characteristics	Category	Frequency (%)
Gender	Male	192 (45.5)
	Female	230 (54.5)
Age	18–34	168 (39.8)
	35–64	196 (46.4)
	≥ 65	58 (13.7)
Marital status	Single	128 (30.3)
	Married	222 (52.6)
	Widowed	51 (12.1)
	Divorced	21 (5)
Area of residence	Rural	306 (72.5)
	Urban	116 (27.5)
Living situation	With families	351 (83.2)
	Alone	67 (15.9)
	Institution	4 (0.9)
Social drug use	Patients with smoking habit	13 (3.1)
	Patients with a drinking habit	191 (45.3)

UoGCSH University of Gondar comprehensive specialized hospital

### Clinical data

The study identified 260 chronic medical conditions and 888 post-admission diagnoses. More than three-fourths (80.8%) of patients were taken care of by their families or institutions during hospitalization. More than one-third (36.7%) of patients had a known history of chronic illness. Hypertension (40%) was the most commonly observed chronic illness among patients and pneumonia (15.09%) was the most frequent diagnosis after admission. Renal impairment was identified in a minimal number (15.87%) of study participants (Table 2).

**Table 2:** The clinical characteristics of patients admitted to the emergency ward at UoGCSH

Variables	Frequency (%)
Patients with psychological problems	23 (5.5)
Patients with linguistic problems	9 (2.1)
Patients with uncorrected/uncorrectable visual impairment	50 (11.8)
Patients with uncorrected/uncorrectable hearing impairment	27 (6.4)
Patients with uncorrected /uncorrectable mobility impairment	30 (7.1)
Patients with renal insufficiency (GFR: < 60 ml/min/1.73 ${\mathbf{m}}^{2}$)	67 (15.9)
Patients with hepatic insufficiency or liver cirrhosis	37 (8.8)
Patients are taken care of by others	341 (80.8)
Patients that have chronic medical conditions	155 (36.7%)
Chronic illness before admission	
Chronic Hypertension	62 (30.09)
Chronic diabetes mellitus	43 (20.87)
Chronic Heart failure	33 (16.02)
Chronic HIV infection	20 (9.70)
Other Ѱ (Chronic conditions)	48 (23.3)
Diagnosis after admission	
Diagnosis – Pneumonia	134 (15.09)
Diagnosis – Hypertension	82 (9.23)
Diagnosis – Heart failure	74 (8.33)
Diagnosis – Diabetes mellitus	60 (6.76)
Diagnosis – Dyspepsia	53 (5.97)
Diagnosis – Malaria	43 (4.84)
Other Ѳ (Diagnosis)	442 (49.77)

Ѱ chronic corplumonale, chronic epilepsy, chronic liver disease, chronic hepatitis, chronic schizophrenia, chronic toxic multinodular goiter, chronic myocardial infarction, chronic ischemic stroke, and chronic asthma

Ѳ bronchiectasis, amebiasis, sepsis, acute kidney injury, hypokalemia, bicytopinia, visceral leishmaniosis, hemiplegia, ischemic stroke, glomerulonephritis, chronic kidney disease, nephrotic syndrome, pancytoinia, hepatitis, corplumonale, peptic ulcer disease, urinary tract infection, tuberculosis, epilepsy, retro viral infection, oral candidiasis, anemia, chronic liver disease, hepatic encephalopathy, pyelonephritis, meningitis, acute gastro enteritis, shock, seizure, schizophrenia, toxic multinodular goiter, snake bite, poisoning, deep vein thrombosis, pulmonary embolism, cancer, asthma, atrial fibrillation, tetany, myocardial infarction, and hyperkalemia

*UoGCSH* University of Gondar comprehensive specialized hospital

More than two-thirds (68.2%) of patients had direct admission to the emergency ward of the UoGCSH. Nearly, one-third (31.8%) of patients were transferred or referred from other health care facilities to UoGCSH. The majority of them (69.4%) were referred from primary hospitals. Of all the study participants, less than half (46%) of patients had a history of hospitalization. Nearly, one-third (29.15%) of participants had been taking medication for chronic disease before admission and were on drug therapy for a median duration of 36 months (IQR: 4–121). One-third (32.9%) of patients had used traditional medicine once in their lifetime. Out of these, “Ye wefe” was the most commonly used traditional medicine, accounting for 83.45%. Above two-thirds (68.2%) of participants had comorbid conditions. Of those patients with comorbidities, less than half (42.0%) of the respondents had two or more comorbid conditions (Table 3).

**Table 3:** The clinical characteristics of patients admitted to the emergency ward at UoGCSH

Variables	Category	Frequencies (%)
Direct admission to the emergency ward	Yes	288 (68.2%)
	No	134 (31.8%)
Transferred from other health facilities	Transferred from primary hospital	93 (69.4%)
	Transferred from private hospital	20 (14.9%)
	Transferred from health centers	21 (15.67%)
Patients who were on medication for chronic disease before admission	Yes	123 (29.15%)
	No	299 (70.85%)
Duration of therapy before admission	≤ 36 month	63 (51.22%)
	> 36 month	60 (48.78%)
Presence of co-morbidities	Yes	288 (68.2)
	No	134 (31.8)
Number of comorbidities	1	167 (57.98)
	> 1	121 (42.01)
Charlson co-morbidity index	1–2	157 (37.2)
	≥ 3	130 (30.8)
	None	135 (32)
Estimated ten years of survival	<96%	181 (42.9)
	≥ 96%	241 (57.1)
History of traditional medication use	Yes	139 (32.9)
	No	283 (67.1)
Types of traditional medications used by patients	“Ye wefe”	116 (83.45)
	“Haregressa”	20 (14.38)
	Other	3 (2.16)
History of hospitalization	Yes	194 (46)
	No	228 (54)
Patients who had used medication for chronic disease before admission	Yes	123 (29.15)
	No	299 (70.85)
The number of chronic disease medications the patient was taking prior to admission	1	43 (10.2)
	≥ 2	80 (19)
	None	299 (70.9)
Duration of therapy while taking medication used for chronic diseases before admission	≤ 36 month	63 (14.9)
	> 36 month	60 (14.2)
Number of days in hospital after admission	≤ 5 days	269 (63.7)
	≥ 6 days	153 (36.3)
Number of medication the patients is using now	≤ 4	264 (62.6)
	≥ 5	158 (37.4)

*UoGCSH* University of Gondar comprehensive specialized hospital

During the study period, 268 medications used by patients before admission for chronic disease were identified. Among the 1,741 medications prescribed after admission, most (38.37%) were taken twice daily. Among medications taken for chronic disease before admission, frusemide (30.08%) was the most commonly used, followed by enalapril (25.2%). After being admitted to the ward, over half (53.8%) of patients received ceftriaxone (Table 4).

**Table 4:** Medications that are taken before and after admission among patients admitted to the emergency ward at UoGCSH

Medications used before admission for chronic disease	Frequencies (%)
Frusemide	37 (13.8)
Enalapril	31 (11.57)
Amlodipine	29 (10.82)
Insulin	23 (8.58)
Antiretroviral therapy	19 (7.09%)
Spironolactone	18 (6.72%)
Atorvastatin	15 (5.59%)
Metformin	14 (5.22%)
Other medications used before admission*	82 (30.6)
Medications used after admission	Frequencies (%)
Ceftriaxone	227 (13.03)
Omeprazole	155 (8.9)
Frusemide	115 (6.6)
Azithromycin	109 (6.26)
Insulin	66 (3.79)
Vancomycin	63 (3.62)
Paracetamol	62 (3.56)
Metoclopramide	55 (3.16)
Unfractionated heparin	51 (2.93)
Artesunate	49 (2.81)
Metronidazole	45 (2.58)
Amlodipine	41 (2.35)
Atorvastatin	39 (2.24)
Potassium chloride	38 (2.18)
Enalapril	38 (2.18)
Tramadol	34 (1.95)
Folic acid	32 (1.84)
Aspirin	32 (1.84)
Ferrous sulphate	31 (1.78)
Ciprofloxacin	30 (1.72)
Dexamethasone	27 (1.55)
Other medications used after admission**	692 (39.77)
Medications taken once per day	600 (34.46)
Medication taken twice per day	668 (38.37)
Medication taken three times per day	265 (15.22)
Medications taken four times per day	48 (2.76)
Medications taken six times per day	6 (0.34)
Medications taken more than six times per day	55 (3.16)
Medications taken when required	99 (5.67)

*Glibinclamide, Phenobarbitone, Phenytoin, Salbutamol, Prednisolone, Hydrochlorothiazide, Metoprolol, Warfarin, Aspirin, Haloperidol, Diazepam, Propranolol, and Propyltyouracil

**Diloxanide, Ceftazidime, Sodium stibogluconate, Paramomicin, Prednisolone, Cyclophosphamide, Calcium gluconate, Metformin, Cyanocobalamin, Albendazole, Antituberclosis drugs, Pyridoxine, Phenytoin, Chloroquine, Cotrimoxazole, Hydrochlorothiazide, Lactulose, Digoxin, Warfarin, Propranolol, Doxycycline, Coartum, Spironolactone, Bisacodyl, Acyclovir, Ampicillin, Tinidazole, Hydrocortisone, Dopamine, Diazepam, Mannitol, *Propylthiouracil, Tetanus Antitoxoid,* Magnesium sulphate, Hydralazine, Cimetidine, Salbutamol, Beclomethasone, Antiretroviral, Clopidogril, Methotrexate, Ibuprofen, Morphine, Hydroxyurea, Allopurinol, and Amoxicillin

*UoGCSH* University of Gondar comprehensive specialized hospital

### Prevalence and pattern of medication errors

The present study included 422 admissions, 1741 medication orders, and 3220 patient days. Medication errors were 116.4 per 100 admissions, 28.2 per 100 medication orders, and 152.5 per 1000 patient days. From the total of study participants, 314 (74.4%) had medication errors [95% CI (70.3–78.4)]. More than half (60.19%) of these patients had at least one medication error.

**Fig. 1:**
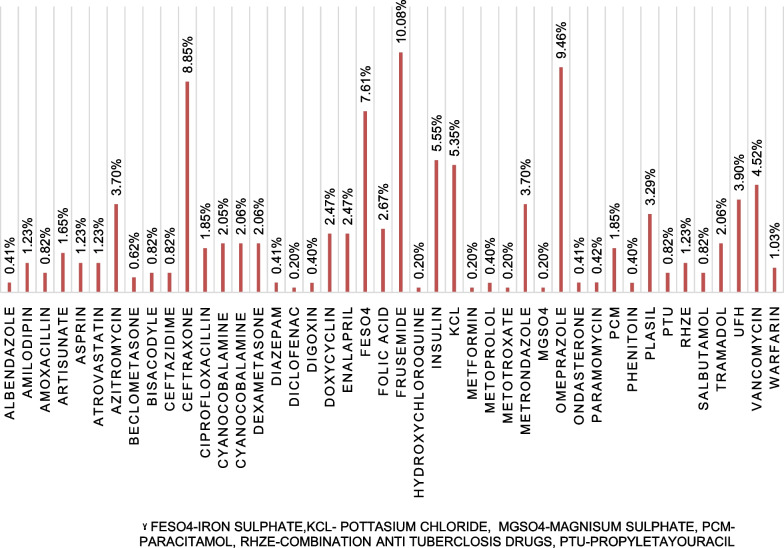
Medications involved in MEs at the emergency ward of UoGCSH

From a total of 486 medications involved in MEs, frusemide (10.08%) was the most common medication, followed by omeprazole (9.46%) and ceftriaxone (8.85%) (Fig. 1).

More than half (61.78%) of patients that encountered MEs were due to prescribers. The most commonly observed (42.35%) stage of ME was the administration stage of medication use by nurses. From a total of 491 MEs identified, the majority (97.75%) were not intercepted or prevented before reaching the patient (Table 5). Omitted dose was the most common type of medication error accounting for 26.27% (Table 6).

**Table 5:** Personnel involved in MEs, stages of MEs and prevention of MEs in the emergency ward at UoGCSH

Personnel involved in MEs	Frequency (%)
Prescribers	194 (61.7)
Nurses	133 (42.35)
Patients or care givers	86 (27.39)
Pharmacists	7 (2.23)
Stages of ME in the medication use process	Frequency (%)
Administration stage by nurses	133 (42.35)
Prescribing stage	117 (37.26)
Administration stage by patient or care giver	86 (27.39)
Transcribing stage	68 (21.66)
Monitoring stage	36 (11.46)
Dispensing stage	7 (2.23)
Prevention of MEs before they occur	Frequency (%)
MEs not intercepted before they reach the patient	480 (97.75)
MEs intercepted before they reach the patient	11 (2.25)

*ME* medication error

UoGCSH University of Gondar comprehensive specialized hospital

**Table 6:** Types of medication error among patients admitted to the emergency ward at UoGCSH

Type of ME in medication use process	Frequency (%)
Dose omitted	129 (26.27)
Omitted transcriptions	117 (23.83)
Wrong dose, strength or frequency	93 (18.94)
Wrong time of dose administration	49 (9.98)
Necessary monitoring not ordered	34 (6.92)
¥ Others	69 (14.05)

¥ Drug given to wrong patient (0.81%), improper drug for an indication (5.29%), drug–drug or drug disease interaction (0.20%), contraindication (0.20%), wrong rate of administration (0.81%), wrong method of administration (0.81%), wrong quantity (0.81%), wrong dosage form (0.81%), wrong preparation method (0.20%), wrong duration (3.46%), and monitoring not performed (0.61%)

*UoGCSH* University of Gondar comprehensive specialized hospital

### Category and patient outcome of medication errors

**Fig. 2:**
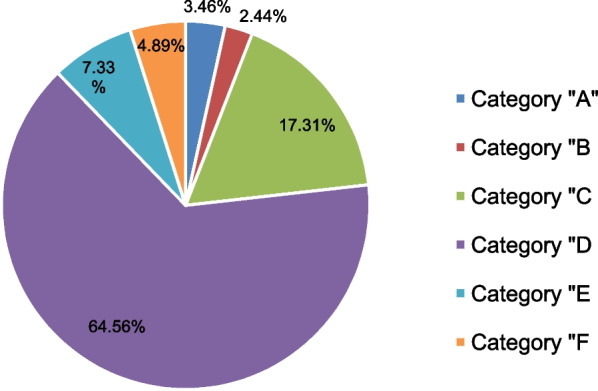
Medication error categories according to the NCC MERP severity index in the emergency ward at UoGCSH

**Fig. 3:**
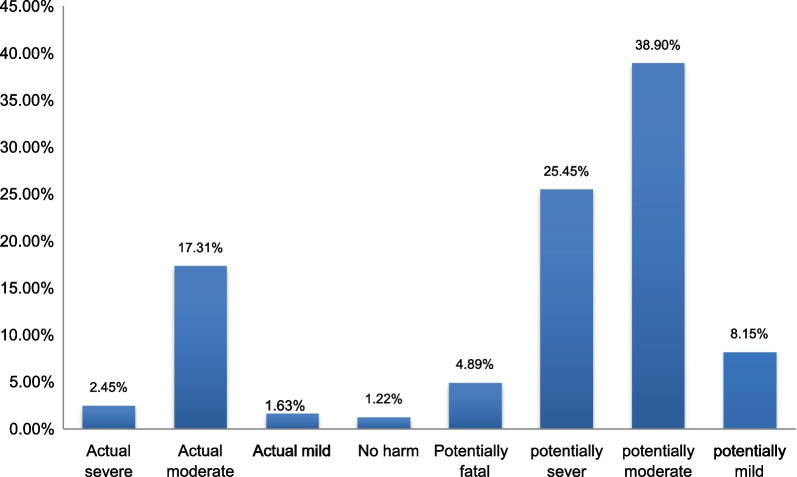
Outcome of event among patients admitted to the emergency ward at UoGCSH

According to the NCC MERP severity index, the majority of medication errors fell into category “D” (64.56%) followed by category “C” (17.31%) (Fig. 2). The most common (38.9%) patient outcome related to medication errors was potentially moderately harmful (Fig. 3).

### Possible causes of medication errors

A total of 443 possible causes of ME were identified. From these, more than half (61.39%) were related to staff factors and less than one fourth (19.86%) were related to patients’ or care givers’ factors. Staff tend to develop ME because of behavioral factors, accounting for 55.15% of possible causes of ME. Patients were more likely to develop ME due to communication factors observed in 39.77% (Table 7).

**Table 7:** The possible cause of medication error among patients admitted to the emergency ward at UoGCSH

Patient factors		Frequency (%)		Frequency (%)
Cognitive factors (*N* = 34)	Perception/understanding	25 (73.53)		
	Knowledge based/problem solving	9 (26.47)	Failure to synthesize/ act on available information	2 (22.22)
			Problem with complexity	7 (77.78)
Performance factors (*N* = 1)	Technical error in execution (physical-skill based)	1 (100)	Lapse error	1 (100)
Behavior factors (N = 8)	Attention issues	6 (75)	Distraction/Inattention	1
			Absent mindedness	5
	Fatigue/exhaustion	2 (25)		
Communication factors (*N* = 35)	Communication method	29 (82.85)	Verbal communication	29 (82.85)
	Communication with staff	6 (17.15)		
Disease related factors (*N* = 2)	International classification of disease	2 (100)		
Emotional factors (*N* = 7)	–	7 (100)		
Social factors (*N* = 30)	–	30 (100)		
Staff factors		Frequency (%)		Frequency (%)
Cognitive factor (*N* = 99)	Perception/understanding	7 (7.07)		
	Knowledge based/problem solving	75 (75.75)	Failure to synthesize/act on available information	66 (88)
			Problems with causality	2 (2.67)
			Problems with complexity	7 (9.33)
	Illusory correlation	3 (3.03)		
	Halo effect	14 (14.14)		
Performance factors (*N* = 111)	Technical error in execution (physical-skill based)	90 (81.08)	Slips error	22 (24.44)
			lapse error	68 (75.56)
	Rule based	3 (2.7)	Misapplication of good rules	2 (66.67)
			Application of bad rules	1 (33.33)
	Selectivity	3 (2.7)		
	Biased reviewing	15 (13.51)		
Behavioral factors (N = 150)	Attention issues	50 (33.33)	Distraction/inattention	33 (66)
			Absent-mindedness	16 (32)
			Over attention	1 (2)
	Fatigue/exhaustion	40 (26.67)		
	Overconfidence	1 (0.67)		
	Non-compliance	55 (36.67)		
Communication factors (*N* = 58)	Communication method	47 (81.03)	Paper based	39 (82.97)
			Electronic	1 (2.13)
			Verbal	7 (14.89)
	Communication with staff	3 (5.17)		
	Communication with patients	8 (13.80)		
Organizational factors (*N* = 82)	Organization of teams	25(100)		
	Resources or workload	57(100)		
External factors (*N* = 1)	Products, technology or infrastructure	1(100)		

*UoGCSH* University of Gondar comprehensive specialized hospital

### Types of medication related interventions given

**Fig. 4:**
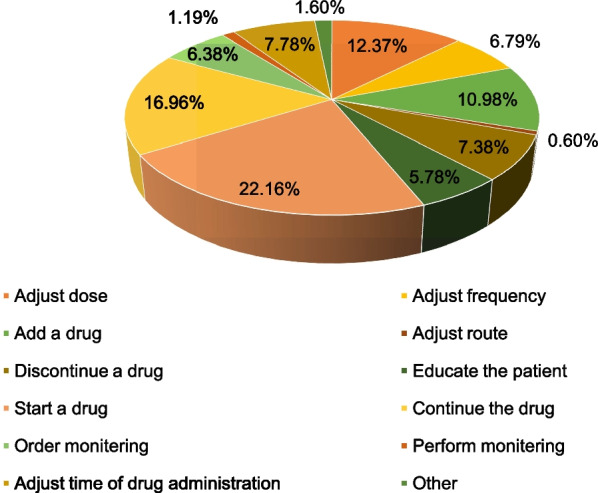
Types of medication-related intervention made among patients admitted to the emergency ward at UoGCSH

During the study, clinical pharmacists gave 501 medication-related interventions. The most common intervention (22.16%) was to start medication (Fig. 4).

### The rate of acceptance of interventions

In the current study, interventions were given to 422 staff and patients or care givers. From these 46.2% were physicians, 31.75% were nurses and 22.04% were patients or care givers.

**Fig. 5:**
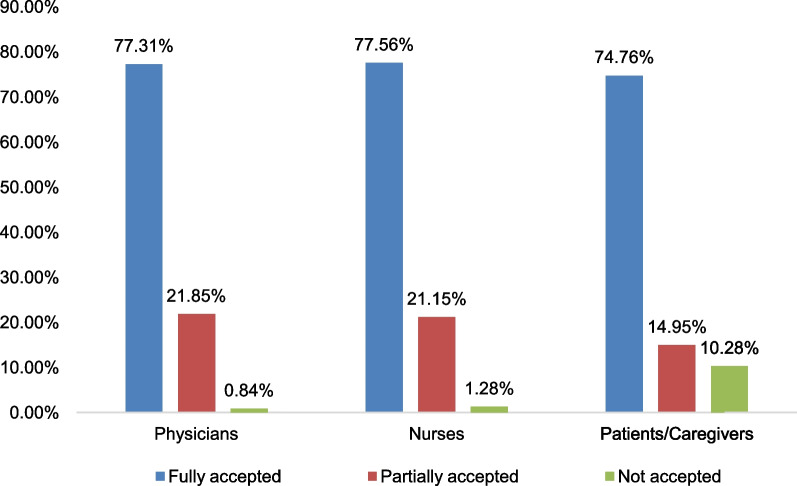
The rate of acceptance to the interventions given in the emergency ward at UoGCSH

Out of 501 interventions, 47.5% were given to physicians followed by nurses, 31.14%, and, finally, patients or caregivers, 21.36%. More than three-fourths (77.31%) of physicians fully accepted the interventions provided by clinical pharmacists (Fig. 5).

### Associated factors with medication error

Multivariable logistic regression analysis results showed that a hospital stay of more than or equal to six days and the use of more than or equal to five medications were significantly associated with ME.

The odds of ME in patients who stayed in the hospital for six or more days were three times higher than those who stayed less than six days; AOR: 3.00 (95% CI 1.65–5.45). The odds of ME in patients with polypharmacy were five times more pronounced than those who took less than five medications; AOR: 5.47 (95% CI 2.77–10.81). The odds of ME in patients with a Charlson comorbidity index value of three or more were two times higher than those with two or less; AOR: 1.94, (95% CI 1.02–3.68) (Table 8).

**Table 8:** Associated factors with medication errors among patients admitted to emergency ward at UoGCSH

Variables	Presence of ME		COR(95%CI)	AOR(95%CI)	*P* value
	Yes	No			
Gender					
Female	178	52	1.41 (0.90–2.18)	1.26 (0.77–2.04)	0.36
Male	136	56	1	1	
Patients with uncorrected/uncorrectable visual impairment					
Yes	42	8	1.93 (0.87–4.25)	1.85 (0.77–4.42)	0.17
No	272	100	1	1	
Patients with uncorrected/uncorrectable renal impairment					
Yes	55	12	1.69 (0.87–3.31)	1.39 (0.66–2.91)	0.38
No	259	96	1	1	
Patients diagnosed with pneumonia					
Yes	109	25	1.76 (1.06–4.16)	1.24 (0.70–2.20)	0.46
No	205	83	1	1	
Presence of comorbidity					
Yes	223	65	1.62 (1.02–2.55)	1.153 (0.66–2.02)	0.62
No	91	43	1	1	0.09
Charlson comorbidity index					
None	112	45	1	1	
1–2	105	25	1.68 (0.96–2.94)	1.09 (0.59–1.99)	0.79
≥ 3	97	38	1.02 (0.61–1.70)	1.94 (1.02–3.68)	**0.04**
Patient stay in the emergency ward after admission					
≥ 6 days	136	17	4.09 (2.32–7.18)	3.00 (1.65–5.45)	** < 0.001**
≤ 5 days	178	91	1	1	
Number of medication the patient is using after admission					
≥5 Drugs	146	12	6.95 (3.66–13.18)	5.47 (2.77–10.81)	** <0.001**
<5 Drugs	168	96	1	1	

Bold values indicate statistical significance

Chi-square value—6.625

*UoGCSH* University of Gondar comprehensive specialized hospital

## Discussion

Medication errors are the most common problem in health care facilities. For several reasons, this phenomenon cannot be avoided, but it can be reduced by identifying possible causes. MEs have a significant negative impact on patients’ safety as well as the economic burden on a given society.

In the present study, the prevalence of medication errors was quite high (74.4%). This finding is consistent with the study conducted in Iran [43]. However, it is higher than what was observed in a study conducted in Ethiopia [10], Uganda [44], and Malaysia [19]. This variation may be due to the study settings. In the present study the study setting was the emergency ward which is more vulnerable to medication error occurrences [45]. The working environment is frustrating, with high patient flow that results in healthcare professionals making errors in the medication use process [46]. Medication errors have a significant burden and attention should be given to ensure safe health care practices.

In the current study, the most common stage of medication error was the administration stage by nurses, which occurred in (42.35%) patients, followed by the prescribing stage, which occurred in (37.26%) patients. Similar findings were observed in the Norway study [47]. On the contrary, a study in Ethiopia [10] and Indonesia [48] found that the prescribing stage was the most common stage of ME. In the emergency ward, most patients need support, are critically ill, and should take medications parenterally for enhanced bioavailability and fast onset of action [49, 50]. For this reason, most medications should be administered by a nurse, and during the process, several medication errors could occur [51]. Attention should be paid to parenteral preparation administration. Such types of preparation are more prone to errors and can harm patients if not handled correctly [52].

Among the 491 MEs that occurred in the emergency ward of the UoGCSH, (97.75%) of them were not intercepted/detected before reaching the patient. This result is similar to a study conducted in Ethiopia [10]. A quite different result was observed in the United States [53] and in Malaysia [19] in which the majority of MEs were caught before they reached the patient. One of the reasons a medication error was not prevented in the hospital is because the emergency ward at UoGCSH receives, diagnoses, and treats patients referred from other primary hospitals, private hospitals, and governmental health centers, which predisposes the workplace environment to stress and an increase in work load [54, 55]. Therefore, professionals working in such an environment may not have enough time to assess and prevent MEs. Clinical pharmacists working on hospital wards could detect MEs and ensure patient safety [56]. Incorporating clinical pharmacists into health care environments under stress would be beneficial.

The current study showed that starting a medication is the most common intervention made by clinical pharmacists, with a value of (22.16%). This finding is in line with a study conducted in Thailand [57]. However, a study done at Alem Ketema Enat Hospital, Ethiopia, revealed that the most common intervention is to discontinue the medication involved in ME [58]. Another study done at Jimma University Medical Center, Ethiopia, found that the most frequent interventions given by pharmacists were to stop a drug and to begin a new drug [59]. Moreover, a study in Turkey revealed that the most common intervention made by pharmacists was monitoring drug therapy (31.0%) [60]. This difference may be due to the fact that patients in the present study needed to start taking medications. However, prescription orders were not transcribed into a prescription paper by physicians (omission of transcription). Thus the patient can’t bring the medication with them to start. In addition, the nurse and the patient or caregiver might not begin to administer or take medication that is already in the hands of the patient (omission of dose). Healthcare professionals should minimize errors of omission in transcription and administration. And patients should immediately start taking prescribed medications.

The current study shows that clinical pharmacists gave 238 interventions to physicians, and (99.14%) were accepted. This finding is consistent with a study done in Saudi Arabia [61]. A study in Sweden [62], the Netherlands [63], and Saudi Arabia [64] also found similar results. Errors in the medication use process should be addressed with appropriate interventions.

The present study showed that staying in the hospital for 6 or more days was 3 times more likely to expose patients to ME than staying less than or equal to 5 days [AOR: 3.00, 95%CI (1.65–5.45), *p* < 0.001]. This finding is consistent with studies done in Ethiopia [10] and Egypt [65]. It can be justified that as patients stay on the ward for extended periods of time, they may be exposed to multiple medications, leading to medication errors [65]. Patients with complicated diseases and/or comorbidities have long hospital stays [66, 67]. During their long hospital stay, an extensive medical assessment will be performed, and additional medications will be prescribed, transcribed, administered and dispensed; depending on the type of drug, monitoring will be ordered and performed [68] and ME might occur at one stage. It can be further clarified that, as the duration of a hospital stay increases, the chance of acquiring a communicable disease will increase, especially in critically ill patients [69, 70]. As a result, for the acquired disease, additional drugs that increase ME risk could be prescribed. Healthcare professionals should pay attention to patients who will spend a long time in health care facilities and ensure medication safety.

The current study found that the number of medications patients used after admission to the emergency ward was significantly associated with ME. Patients with polypharmacy after admission experienced nearly 5 times more ME than patients who took < 5 medications [AOR: 5.47, 95% CI (2.77–10.81), *p* < 0.001]. This finding is in line with a study in Ethiopia [10], Egypt [71], Pakistan [72], and Japan [73]. It can be stated that multiple drug therapy could result in ME and subsequent harm, which is also supported by a wide number of publications. If possible, the use of multiple medications should be minimized. Patients with polypharmacy should be closely followed throughout the medication use process.

Furthermore, the present study revealed that patients with a Charlson comorbidity index value of three or above experienced 2 times more ME than patients with a Charlson comorbidity index value of two or less [AOR: 1.94, 95% CI (1.02–3.68), *p* < 0.04]. Studies conducted previously showed that the presence of comorbidities was associated with MEs [72, 74]. Several medications were prescribed to patients with comorbidities in the current study. MEs can occur at one or more stages as the number of medications prescribed increases. Healthcare professionals and patients should pay attention to medication use in patients with a large number of comorbidities.

## The strength and limitations of the study

The authors put their maximum effort into presenting the magnitude, types, categories, possible causes, and patients’ outcomes of MEs. In addition, the names of medications involved in MEs, associated factors for MEs, types of medication-related interventions given, and the rate of acceptance of the interventions were also revealed. However, this study has certain limitations. In the event of a medication error, intervention was provided to the person or situation that was responsible. This might affect the reoccurrence of some MEs and reduce their actual prevalence during the study period. In addition, even if patients’ chart records were used to obtain MEs that occurred during the night, the exact figure of medication error occurrence might not be observed. Since the data collectors were not in the ward at night time. In addition, healthcare professionals working at the emergency ward were aware of the study, which could affect the incidence of events of interest (MEs). Furthermore, the study is single-centered and might not be generalized to other hospitals in Ethiopia.

## Conclusions

Almost three-fourths of adult patients admitted to the emergency ward encountered at least one ME. Starting medications was the major intervention given by clinical pharmacists. Most of the interventions offered were accepted. Medication errors had a significant association with length of hospital stay, polypharmacy, and a Charlson comorbidity index value of three or greater. The prescribing of multiple medications to patients in the emergency ward of UoGCSH should be taken into account. Patients who stay in the hospital for a longer period of time and patients with an increased number of comorbidities should be closely monitored since these patients could be more prone to MEs. Healthcare professionals need to pay attention to and be compliant with the correct medication use practice. The UoGCSH should pay attention to any circumstances that cause distraction or interruption in medication prescribing and administration on the emergency ward. Furthermore healthcare workers' fatigue and exhaustion should be controlled by reducing working hours and boosting staff rotations in the organization.

## Data Availability

The data sets used and analyzed during the current study are available from the corresponding author on reasonable request.

## Abbreviations

ADE: Adverse drug event
CCI: Charlson comorbidity index
eGFR: Estimated glomerular filtration rate
ICU: Intensive care unit
IQR: Interquartile range
ME: Medication error
NCC MERP: National Coordinating Council for Medication Error Reporting and Prevention
UoGCSH: University of Gondar Comprehensive Specialized Hospital
WHO: World Health Organization
